# Season of birth and schizotypy in a sample of undergraduate students

**DOI:** 10.1007/s00127-024-02719-w

**Published:** 2024-07-09

**Authors:** Andrei Szöke, Jean-Romain Richard, Maria Ladea, Aziz Ferchiou, Elie Ouaknine, Victor Alexandru Briciu, Mihail Cristian Pirlog, Mihai Bran, Baptiste Pignon, Franck Schürhoff

**Affiliations:** 1 Univ Paris Est Creteil, INSERM, IMRB, AP-HP, Hôpitaux Universitaires “H. Mondor”, DMU IMPACT, Fondation Fondamental, 94010 Creteil, France; 2 Univ Paris Est Creteil, INSERM, IMRB, Fondation Fondamental, 94010 Creteil, France; 3 AP-HP, Hôpitaux Universitaires “H. Mondor”, DMU IMPACT, 94010 Creteil, France; 4 Faculty of Sociology and Communication, Department of Social Sciences and Communication, Transilvania University of Brasov, Brasov, Romania; 5 Department of Medical Sociology, Faculty of Medicine, University of Medicine and Pharmacy of Craiova, Craiova, Romania; 6 Coltea Hospital, Bucharest, Romania

**Keywords:** Schizophrenia, Psychosis, Urbanicity, Risk factors

## Abstract

**Purpose:**

In line with the psychotic continuum theory, the study of psychometric schizotypy in non-clinical samples has been proposed as a convenient yet powerful method for studying the etiology of psychosis. Based on this paradigm, several studies explored the association between season of birth (SoB) and schizotypy but led to inconsistent results. Building on the analysis of the previous studies, in the present study, we aimed to advance our understanding by improving the methodology (using a homogeneous group, eliminating unreliable respondents, taking into account potential confounders) and the reporting.

**Methods:**

Subjects were recruited among undergraduate students from 3 Romanian Universities. To limit the potential influence of invalid response, we applied methods for detecting unreliable and/or biased questionnaires and excluded subjects with unreliable/ biased answers from the analyses. Schizotypal dimensions were measured using the Romanian translation of the 22-items Schizotypal Personality Questionnaire-Brief (SPQ-B). The association between schizotypy scores and season of birth was explored using linear regression.

**Results:**

In a sample of 484 undergraduate students from Romania, we found that being born in late winter/early spring (February and March) was associated to higher total schizotypy score and disorganization. Furthermore, we found that restricting the sample to subjects born in an urban environment increased the strength of the association.

**Conclusion:**

This study is consistent with an association between SoB and the risk of psychotic disorders.

**Supplementary Information:**

The online version contains supplementary material available at 10.1007/s00127-024-02719-w.

## Introduction

Psychotic disorders are major causes of suffering for patients and their families, are major contributors to years of life lost and years lost due to disability and have huge individual and societal costs [1, 2]. As a consequence, significant efforts to understand their etiology and to develop adequate treatments and preventive measures have been deployed. This is reflected in the growing number of publications on the subject over the last decades. However, to date, the research has been only (very) partially successful.

As our understanding progressed, 2 paradigms emerged for the research. First, that of a psychotic continuum at both clinical and etiological levels [3–6]. At clinical level, this means that differences between severe psychotic disorders (e.g. schizophrenia), mild and subclinical psychosis (e.g. schizotypy) and isolated psychotic manifestations in nonclinical populations are all different degrees of a qualitatively similar category. On the other hand, the etiological continuum implies the existence of similar etiologies all along the psychotic continuum, with differences being of degree and not of nature. The second, more general, paradigm is that of risk factors (RF) which are at the origins of complex, and most often chronic disorders among which psychotic disorders. By contrast to simpler direct causes, as seen for example in infectious diseases, risk factors are neither necessary (are not present in all ill subjects) nor sufficient (they might be present, without leading to the disorder) [7].

There are several advantages of studying subclinical, quantitative psychotic traits in the general population instead of case–control studies of schizophrenia, e.g. no interference from post-diagnosis factors like treatment and hospitalization, less risk of errors of classification/diagnosis, better stability and better statistical power (see also Barrantes-Vidal et al. [8]). Because of these advantages, and based on the two paradigms enunciated above, a quantitatively significant research of the association of a schizotypal dimensions with putative RF for schizophrenia emerged.

One such factor that has been associated with risk for schizophrenia is season of birth (SoB) [9–11]. Indeed, birth in winter has been associated with a slight increase in the risk for developing schizophrenia (RR = 1.04–1.07). Two points are important in understanding the link between SoB and schizophrenia. First: a sizeable part of the population is exposed to the potential risk period which makes it an important risk factor at population level. The second point is that, as is the case for several “risk factors” for schizophrenia, the SoB is only a marker for one (or several) yet unknown risk factor(s). Several hypotheses have been proposed to explain how SoB might be related to schizophrenia. An explanatory hypothesis of the role of SoB in the risk for schizophrenia has to provide a mechanistic link between factors that show seasonal variation and (early) neurodevelopment. The list of seasonal (putative) risk factors is large. It includes factors that are directly linked to the seasons (duration of the day-light, temperature, rain) or indirectly (seasonal infections, nutrition, vitamin D, air pollution patterns) [12]. Some of these factors will directly influence the child (mainly after birth) and others will influence his/her health indirectly by affecting maternal health/physiology (mainly before delivery). That SoB is linked to neurodevelopment is evident not only from data on risk for schizophrenia [9, 10] and autism [13] but also from risk for neurological disorders like multiple sclerosis [14] and even more directly from risk for pediatric cerebral tumors [15] and neural tube defects [16]. Despite the high number of putative risk factors and the numerous potential outcomes the mechanism that link them are probably limited. Aside from direct influences on development (e.g. nutrition) indirect effects via inflammatory mechanisms or by epigenetic changes have been proposed (for infections, air-pollution, vitamin D) [17, 18]. These hypotheses have not yet been tested in case–control studies involving subjects with schizophrenia because of the practical and statistical (i.e. power) limitations alluded before. Thus, the demonstration of a similar association with schizotypy (or specific schizotypal dimensions) in the general population is important and might pave the road to test these hypotheses.

Although at least 8 studies of the association of SoB with psychometric schizotypy have been published to date (see Konrath et al. [19] for a summary of the published studies—to which the study by Mimarakis et al. [20] has to be added), a clear picture has not yet emerged. There were positive findings either for total schizotypy [19, 21] or specific dimensions/traits [19, 20, 22, 23] but also negative findings (i.e. no differences in scores according to SoB) [24, 25]. Furthermore, the significant associations were not always the same i.e. in different studies increased scores were not always associated with the same season or month. This is not so surprising given the fact that definition of the SoB varied widely (e.g. meteorological or astronomical season, half years with different starting points, or months) and also did the measures of schizotypy (different versions of the SPQ, one or several of the Chapmans’ questionnaires of magical ideation, perceptual aberrations or physical anhedonia). Also, the dimensions analyzed and reported varied from study to study (total score, positive or negative dimension, etc.). Furthermore, important characteristics of the samples – that might confound the association – differed between studies, e.g. age of subjects (mean and range), latitude and climate of birth place etc. Finally, important confounders (including substance abuse data, unreliable style of responding, urbanicity of place of birth [26–28]) were not always measured or used in modeling the link between SoB and schizotypy. An additional problem was that even when some of the variables of interest were available (e.g. the different dimensions of the SPQ), they were not always reported thus making comparison of the results impossible (e.g. Cohen and Najolia [25]).

In this context, the study by Konrath et al. [19], represents, in our view, a step forward. Indeed not only this is – by far – the largest study but, by reporting results for total and dimension scores, by month and also using different definitions of SoB, it allows for comparison. In this study—of more than eight thousand subjects and using different definitions of SoB—the only group that showed significantly increased scores for total schizotypy was that of subjects born in late winter and early spring (i.e. during the months of February and March). Positive (i.e. cognitive-perceptual) and disorganization dimensions also were significantly increased in this group. All comparisons were adjusted for sex and age. Among the limitations of this study were the age range of participants which was very large (18–104), the absence of a test to identify invalid responding and the fact that no other potential confounders (substance use, education etc.) were used.

Thus, in the present study, our aim was to replicate and extend the findings from Konrath’s et al. study [19] by collecting similar variables of interest and using a similar reporting procedure but also improving the design by taking into account the risk of inaccurate responding and by reducing the heterogeneity introduced by a large range of ages, years of birth and education. As such, we made the hypothesis of statistically significant higher scores in subjects born in February and March (compared with the rest of participants) for the total score but also for positive and disorganized dimensions. As some of the explanatory hypotheses of the association between SoB and schizotypy suggest an interaction between SoB and urbanicity (e.g. sun exposure and vitamin D deficit, infectious disease risk), we also repeated the analyses in a sample restricted to subjects born in an urban setting. We hypothesized that in this sample the differences between those born in late winter-early spring (February–March) and the rest of the sample will be more important.

## Methods

### Subjects

Subjects were recruited among undergraduate students from 3 Romanian Universities: Bucharest Medical School, Craiova Medical School and Brasov (Sociology). Details about the recruitment process are provided elsewhere [29]. In short, the subjects were informed of the aim of the study (i.e. to assess personality traits and their relation to demographic variables and substance consumption), of the fact that the questionnaires were anonymous, and of the necessity of answering all the questions as best and as honestly that they can. They were also informed that the study has been approved by an ethics committee and that they were free to participate in the survey or to decline.

Among the available subjects, we operated a selection in order to enhance homogeneity and limit statistical noise. A negative correlation between age and schizotypy scores has been previously described [30]. In order to enhance homogeneity of the sample and limit the variability in schizotypy scores for reasons other than the season of birth, we decided to limit the analyses to subjects younger than 30 years.

To limit the potential influence of invalid response, we applied methods for detecting unreliable and/or biased questionnaires and excluded subjects with unreliable/ biased answers from the analyses. To this aim, we added 3 validity items and 5 social desirability items randomly distributed among the 22 SPQ-B original items (see details below). Subjects with one (or more) invalid answer(s) were classified as unreliable and were thus excluded from the analyses as were the subjects showing a social desirability score that exceeded the sample mean plus two standard deviations.

### Data recorded

Sociodemographic data collected included gender, age, date and place (urban vs. rural) of birth. Several questions explored consumption of psychoactive substances (no details on the specific substance), alcohol and tobacco. The corresponding variables were dichotomized according to frequency of consumption. Answers were considered as positive when corresponding to at least once a week for alcohol, daily consumption for tobacco and at least “occasional” for psychoactive substances.

Several variables related to the concept of “season of birth” (see introduction) have been derived from the date of birth: month of birth (as reported by the subjects), astronomical season of birth (i.e. winter from December 21st to March 20th, spring from March 21st to June 20th, summer from June 21st to September 20th and fall from September 21st to December 20th), meteorological season of birth (i.e. winter from December 1st to end of February, spring from March 1st to May 31st, summer from Jun 1st to August 31st and fall from September 1st to December 31st) and a high risk period (February and March) based on the study by Konrath et al. [19].

Schizotypal dimensions were measured using the Romanian translation of the 22-items Schizotypal Personality Questionnaire-Brief (SPQ-B), in a Likert format (from 1 = completely disagree to 5 = completely agree). Total score was calculated by adding the scores for each of the 22 items (range 22–110). Dimensions scores were also calculated for positive (10 items), negative (5 items) and disorganized (7 items) dimensions (for more details see Ladea et al. [29]). To the 22 items questionnaire we added 3 validity and 5 social desirability items. Two of the validity items were items for which some of the responses are very improbable (infrequent answers). The first one was “Sometimes I feel tired or sleepy” (infrequent answer “completely disagree”) and the second one “I have answered this questionnaire the best I could” (infrequent answers—“completely disagree” or “disagree”). Such items have previously been shown to capture invalid responses [31, 32]. The third validity item, “I find it easy to communicate clearly what I want to say to people”, has the exact opposite meaning of one of the SPQ items (*i.e.*, “I find it hard to communicate clearly what I want to say to people”). Answers of the same polarity (*i.e.*, scores of 1 or 2, or scores of 4 or 5) to both of those items were considered indicative of unreliable responding. To eliminate biased questionnaires due to social desirability we used 5 items extracted from the Eysenck’s Lie scale (see Ladea et al. [29] and the supplementary material for details). A desirability score was calculated by summing the 5 items and questionnaires for which this score exceeded the sample mean plus two standard deviations were considered biased and excluded from further analyses.

### Statistical analyses

The main analyses (of total and for each of the three schizotypy scores) concerned the risk period described by Konrath et al. [19] (February–March). We also conducted secondary analyses using the alternative definitions of the season of birth. Also, we repeated these analyses in a sample restricted to subjects born in an urban environment. The main reasons for repeating the analyses in the sample of urban born subjects (as opposed to using a more complex multivariate model) were the small numbers of subjects born in a rural environment and the more straightforward interpretation of results.

To describe our sample, we used the following variables: demographic, substance consumption, SPQ scores. For continuous variables, we calculated mean and standard deviation and for categorical variables number and percentage. We also calculated the mean and standard deviation for the SPQ score according to month of birth.

Univariable analyses: the association between the different definitions of season of birth and schizotypy scores was assessed using Student’s t. We also investigated the association of schizotypal scores with (putative) confounders i.e. demographic variables (sex, age, place of birth) and substance consumption using Student’s t or ANOVA statistics (for categorical variables) and correlations (Pearson’s) for continuous variables.

The association between schizotypy scores and season of birth was also explored in multivariable analyses using two different models of linear regression. In the first model, only age and gender were included as potential confounders. In the second model, to these demographic variables, data on tobacco and psychoactive substance consumption were added.

All analyses were done using R version 4.3.0 (2023-04-21 ucrt) – “Already Tomorrow”, Copyright (C) 2023 The R Foundation for Statistical Computing, Platform: x86_64-w64-mingw32/×64 (64-bit).

## Results

### Descriptive statistics

The initial sample consisted of 580 subjects. Of these 10 had incomplete data for SPQ or birth date and 29 were not considered because they were older than 30. Of those eligible, 47 had at least one invalid answer and 10 had desirability scores exceeding the threshold, and were thus excluded of the subsequent analyses.

After applying all these exclusion criteria, our sample consisted of 484 subjects. Among those most were women, urban born and single. The mean age was 24. Detailed demographic data, data about substance consumption and SPQ-B scores are provided in Table 1. Total scores according to month of birth are shown in Table 2 (and in Fig. 1 in the supplementary material).

**Table 1:** Description of the sample

Total	N	484
Gender (F)	N (%)	374 (77.27%)
Age	Mean (SD)	23.76 (1.83)
Tobacco	N (%)	235 (48.6%)
Alcohol	N (%)	156 (32.2%)
Psychoactive substances	N (%)	58 (12.0%)
Urban birth	N (%)	453 (93.6%)
Single	N (%)	318 (65.7%)
SPQ scores total	Mean (SD)	54.85 (11.75)
Positive	Mean (SD)	25.39 (6.28)
Negative	Mean (SD)	15.87 (4.40)
Disorganization	Mean (SD)	13.59 (4.11)

**Table 2:** SPQ total scores and sub-scores by month of birth

N		Jan	Feb	Mar	Apr	May	June	July	Aug	Sep	Oct	Nov	Dec
		43	38	41	43	50	42	39	42	32	41	35	38
SPQ total	Mean	53.67	59.08	57.39	55.42	54.14	53.19	53.87	53.69	53.63	56.34	54.91	52.97
	SD	12.61	10.70	11.46	11.14	11.77	12.97	12.15	12.53	11.21	12.43	11.08	10.14
	t^a^	0.19	2.24	1.63	0.87	0.39	Ref	0.26	0.20	0.16	1.22	0.64	−0.8
	p^a^	0.85	**0.026**	0.10	0.38	0.70	Ref	0.79	0.85	0.87	0.22	0.52	0.93
Positive	Mean	25.19	26.66	26.59	25.58	25.20	24.40	24.97	24.79	25.19	26.24	25.60	24.29
	SD	7.09	6.20	6.72	6.13	6.39	6.06	5.63	6.16	5.19	6.87	6.63	6.11
	t^a^	0.57	1.60	1.58	0.86	0.60	Ref	0.41	0.28	0.53	1.33	0.83	−0.08
	p^a^	0.57	0.11	0.12	0.39	0.55	Ref	0.69	0.78	0.60	0.19	0.41	0.94
Negative	Mean	15.77	16.95	16.49	15.88	15.50	15.52	15.44	16.17	15.13	16.32	16.03	15.26
	SD	4.23	4.60	4.16	4.45	4.17	5.27	4.77	4.54	4.46	4.26	4.12	3.85
	t^a^	0.25	1.44	0.99	0.38	−0.03	Ref	−0.09	0.67	−-0.38	0.82	0.50	−0.26
	p^a^	0.80	0.15	0.32	0.71	0.98	Ref	0.93	0.51	0.70	0.41	0.62	0.79
Disorg	Mean	12.72	15.47	14.32	13.95	13.44	13.26	13.46	12.74	13.31	13.78	13.29	13.42
	SD	4.40	3.64	3.91	3.98	3.70	4.38	4.26	4.17	3.91	4.38	4.12	4.19
	t^a^	−0.61	2.41	1.17	0.78	0.21	Ref	0.22	−0.59	0.053	0.58	0.025	0.17
	p^a^	0.54	**0.016**	0.24	0.44	0.84	Ref	0.83	0.56	0.96	0.56	0.98	0.86

^a^For each month t statistics (Student test) and p for comparison with the reference month (June); in bold characters p < 0.05

### Univariable analyses

The association between the SPQ-B scores (total and the three dimensions) and individual variables of interest i.e. SoB and potential confounders are presented in Table 3. The subjects born in the “risk period” showed an increase in all the SPQ-B scores. However, the difference reached significance only for the total score and disorganization. The other characteristics that were associated with (significantly) higher scores were psychoactive substances (all scores but positive) and tobacco (all scores but negative) use, as well as younger age (all scores but negative). No significant differences were associated with alcohol consumption; sex significantly influenced only disorganization (larger scores in males) and urban birth the positive dimension (smaller scores in those born in urban environment).

**Table 3:** Influence of (putative) risk factors and demographic variables on SPQ scores

Variable	Category^a^	SPQ score (Mean)	stat^b^	p-value^c^	Positive	stat^b^	p-value^c^	Negative	stat^b^	p-value^c^	Disorganization	stat^b^	p-value^c^
At risk period^d^	Y	58.2			26.62			16.71			14.87		
	N	54.2	2.79	**0.0054**	25.15	1.92	0.056	15.71	1.85	0.07	13.34	3.065	**0.0023**
Sex	F	54.83			25.52			15.94			13.37		
	M	54.9	−0.05	0.96	24.92	0.89	0.37	15.64	0.64	0.52	14.35	−2.21	**0.028**
Tobacco	Y	56.37			26.27			15.83			14.27		
	N	53.41	2.79	**0.0054**	24.55	3.04	**0.0025**	15.92	−0.21	0.83	12.94	3.60	**0.0004**
Alcohol	Y	54.54			24.94			15.59			14.01		
	N	55.00	−0.4	0.69	25.60	−1.09	0.28	16.01	−0.98	0.33	13.39	1.57	0.12
Psychoactive substances	Y	58.86			26.84			17.10			14.91		
	N	54.30	2.79	**0.0055**	25.19	1.89	0.059	15.71	2.28	**0.023**	13.41	2.63	**0.0087**
Urban	Y	54.66			25.24			15.85			13.58		
	N	57.55	−1.32	0.186	27.58	−2.02	**0.044**	16.19	−0.42	0.68	13.77	−0.26	0.80
Age^3^	-		−0.19	** < 0.0001**		−0.24	** < 0.0001**		−0.04	0.39		−0.14	**0.0021**

^a^Y – (putative) risk factor present/ N – (putative) risk factor absent

^b^t statistics (Student’s t-test) except for Age (correlation coefficient)

^c^Probability of the null hypothesis (i.e. no difference between categories or correlation coefficient not different from 0); in bold characters p <0,05

^d^February–March

### Multivariable analyses

The results from the 2 models (the 1st including only age and gender and the 2nd including also tobacco and psychoactive substances) are similar to the results from univariate analyses showing a significant increase in schizotypy (total score) and disorganization for the subjects born in the “at risk period” compared with the rest of the subjects (see Table 4).

**Table 4:** Associations of SPQ-B total score and sub-scores with season of birth in multivariable analyses (multiple linear regression models)

		Tobacco (ref^a^ No)		Psychoactive subst. (ref^a^ No)		Age		Sex (ref^a^ Male)		At risk period (ref^a^ No)		Model	
		Reg. Coeff	p-value	Reg. Coeff	p-value	Reg. Coeff	p-value	Reg. Coeff	p-value	Reg. Coeff	p-value^d^	R^2^	p-value^d^
Total SPQ score	Model 1^b^	*NA*	*NA*	*NA*	*NA*	−1.17	4.8*10^**–5**^	−0.439	0.73	3.67	**0.010**	0.043	2.116 * 10^**–5**^
	Model 2^c^	1.37	0.22	1.32	**0.0046**	−1.11	2.97*10^**–4**^	0.332	0.79	3.37	**0.017**	0.060	1.806 * 10^**–6**^
Positive dimension	Model 1^b^	*NA*	*NA*	*NA*	*NA*	−0.79	Type="Bold">2.6 * 10^–7^	−0.45	0.503	1.18	0.12	0.056	9.34 * 10^**–7**^
	Model 2^c^	0.82	0.17	0.49	0.050	−0.74	Type="Bold">6.1 * 10^–6^	−0.45	0.24	1.07	0.16	0.064	Type="Bold">5.81 * 10^Type="Bold">–7^
Negative dimension	Model 1^b^	*NA*	*NA*	*NA*	*NA*	−0.08	0.46	0.23	0.63	0.95	0.081	0.0025	0.24
	Model 2^c^	−0.29	0.50	0.45	**0.012**	−0.13	0.27	0.34	0.48	0.85	0.12	0.012	0.056
Disorgani-zation	Model 1^b^	*NA*	*NA*	*NA*	*NA*	−0.30	**0.0030**	−1.12	0.011	1.54	**0.0020**	0.043	2.26 * 10^**–5**^
	Model 2^c^	0.84	0.33	0.38	**0.019**	−0.24	**0.025**	−0.802	0.071	1.45	**0.0034**	0.061	1.27 * 10^**–6**^

*p* values <0.05 are in bold

^a^‘ref’ = reference category: ‘M’ = male; ‘No’ = risk factor absent

^b^Model 1 includes only demographic variables (Age and Sex) and Season of Birth

^c^Model 2 includes demographic variables (Age and Sex), tobacco, psychoactive substances and Season of Birth

### Analyses in the subsample restricted to subjects born in an urban environment

In univariable analyses total, positive and disorganization scores were significantly elevated in the sample of subjects at risk (born February–March). Negative dimension was also elevated (and nearly reached significance with *p* = 0.050). After adjusting for demographic variables (model 1) and demographic and substance consumption (model 2) only total and disorganization scores retained significance (see Table 5).

**Table 5:** Associations of SPQ-B total score and sub-scores with Season of Birth in univariate and multivariable analyses (multiple linear regression models) in the sample restricted to urban born subjects

		Tobacco (ref^a^ No)		Psychoactive subst. (ref^a^ No)			Age		Sex (ref^a^ Male)		At risk period (ref. Feb_Mar)		Model	
		Reg. Coeff	p-value	Reg. Coeff	p-value	Reg. Coeff		p-value	Reg. Coeff	p-value	Reg. Coeff	p-value	R^2^	p-value
Total SPQ score	Univar	*NA*	*NA*	*NA*	*NA*	*NA*		*NA*	*NA*	*NA*	4.31	0.0032	0.019	0.0032
	Model 1^b^	*NA*	*NA*	*NA*	*NA*	−1.18		9.2 * 10^**–5**^	−0.43	0.74	3.92	**0.0068**	0.046	2.41* 10^**–5**^
	Model 2^c^	1.47	0.20	1.37	**0.003**	−1.11		4.3 * 10^**–4**^	0.39	0.76	3.59	**0.012**	0.066	1.19 * 10^**–6**^
Positive dimension	Univar	*NA*	*NA*	*NA*	*NA*	*NA*		*NA*	*NA*	*NA*	1.65	0.036	0.0097	0.036
	Model 1^b^	*NA*	*NA*	*NA*	*NA*	−0.78		1.6 * 10^**–6**^	−0.58	0.39	1.30	0.091	0.055	2.93 * 10^**–6**^
	Model 2^c^	0.83	0.17	0.54	**0.029**	−0.73		1.8* 10^**–5**^	0.95	0.17	1.17	0.13	0.066	1.1 * 10^**–6**^
Negative dimension	Univar	*NA*	*NA*	*NA*	*NA*	*NA*		*NA*	*NA*	*NA*	1.08	0.050	0.0085	0.050
	Model 1^b^	*NA*	*NA*	*NA*	*NA*	−0.11		0.34	0.22	0.66	1.016	0.067	0.0043	0.18
	Model 2^c^	−0.19	0.67	0.46	**0.010**	−0.14		0.23	0.36	0.47	0.91	0.10	0.015	**0.040**
Disorganization	Univar	*NA*	*NA*	*NA*	*NA*	*NA*		*NA*	*NA*	*NA*	1.58	0.0022	0.021	0.0022
	Model 1^b^	*NA*	*NA*	*NA*	*NA*	−0.30		**0.0055**	−1.23	**0.0068**	1.60	**0.0018**	0.046	2.55 * 10^**–5**^
	Model 2^c^	0.82	**0.042**	0.39	**0.019**	−0.24		**0.031**	−0.92	**0.044**	1.51	**0.0031**	0.064	1.80 * 10^**–6**^

*p* values <0.05 are in bold

^a^‘Ref’ = reference category for multivariable models: ‘M’ = male; ‘No’ = risk factor absent

^b^Model 1 includes only demographic variables (Age and Sex) and Season of Birth

^c^Model 2 includes demographic variables (Age and Sex), tobacco, psychoactive substances and Season of Birth

### Analyses of the SPQ scores using different definitions of the SoB

We compared groups of subjects born in winter using either an astronomical definition (three months, beginning on the 21st of December) or a meteorological definition (three months each, beginning with the 1st of December). Using these alternative definitions we did not observe any significant association (in univariate or multivariate analyses) in the whole sample (see supplementary material tables 1S–6S).

## Discussion

The main result of our study is the replication of the earlier finding of increased schizotypy in subjects born in late winter/early spring, i.e. in the months of February and March. Additional analyses showed that this was true not only for the total score of schizotypy but also for the three dimensions (positive, negative, disorganized) although it reached significance only for disorganization. Using different, previously used, definitions for season of birth, we did not find any other significant association. Finally, restricting the sample to subjects born in an urban environment did improve our capacity to detect significant differences. In this restricted sample, in addition to the differences already observed in the total sample, in univariable analyses the positive dimension was significantly increased in the subjects born in the risk period.

It is important to note, before any interpretation of results involving season of birth, that date of birth is only a marker/ a proxy for a risk factor (or several risk factors) yet to be uncovered. Several hypotheses have been formulated on the nature of this (these) factor(s). Some are about characteristics directly depending on the season like day-light duration or temperature. Others are indirectly linked to such characteristics like vitamin D and nutrition or risk of infectious diseases. Furthermore, these characteristics are not uniformly shared in all populations/places depending on the latitude, but also on the situation of the specific place (altitude, distance from the sea etc.) which influence the climate (see classification of climates in Beck et al. [33]). Finally, even for a given population and place, there might also be yearly variations (for example in monthly average temperature or months of heightened influenza risk, etc.). Another important point is that, although for convenience we use the date of birth as a variable of interest, it is highly possible that the time at which the specific (direct, effective) risk factor acts is either before (e.g. in the second trimester of gestation) or after birth (e.g. in the 1 year of life).

Regarding the replication of findings by Konrath et al., we observed a similar pattern of increased schizotypy not only for the same months (February/ March) but also for the total score and (at least in the sample limited to urban born subjects) for positive and disorganized dimensions. As in Konrath’s and al. study the negative dimension was not (significantly) associated with birth in the period of risk [19]. Also, similar to Konrath’s study, we did not observe significant differences in groups defined by SoB according to classical (i.e. three month long) definitions of seasons. Regarding this almost perfect replication of an earlier study, it is important to stress that our study took place at a similar latitude and in the same general climate (mainly continental Dfb in the Köppen-Geiger classification – see Beck et al. [33]). This might be an important point explaining the differences in results with other previous studies.

Another factor that might explain our capacity to uncover this association was the fact that we limited the range of birth years thus reducing potential variation in some of the characteristics that might change yearly. For the same reason (limiting unwanted variation), we used specific methods to identify and eliminate invalid questionnaires.

Although tobacco and psychoactive substance consumption were associated with scores of schizotypy (which was expected given previous studies [34, 35]) they did not change the association between schizotypy scores and season of birth. By contrast, interestingly, restricting the sample to urban-born subjects seemed to improve our capacity of detecting association between SoB and schizotypy.

Our study has several limitations. Compared to some of the previous studies (e.g. Konrath et al. [19]), our sample size is relatively small. However, this has been in part compensated by measures to improve homogeneity and limit the influence of potential confounders (see above). Also, our models explained little of the variation in the SPQ measures (between 4 and 6%—see Table 4). This is similar to other studies on the subject (e.g. Cordova-Palomera et al. [24] found that a model including demographic variables only explained 5% of the variation). However, this result is not surprising because explaining the variation in schizotypy was not the aim of our study and as such we did not measured and include in our model several factors that might have a large influence on schizotypy scores (e.g. genetic/familial factors, recent stressors etc.). Also, the small variation explained by the global model does not change the significance of our findings i.e. a significant association between SoB and schizotypy. Although we inquired about psychoactive substance use, there was no objective test and no detail on the specific substance used. The percentage of subjects that used psychoactive substances in the general population of Romania in 2016 was of 7.9% and most of them used cannabis (5.8%) according to National Report for drug consumption in Romania 2017 [36]. For most substances, these figures are almost double in the 15–34 age band (e.g. 10% for life-time cannabis consumption) which suggests that the prevalence we observed (12%) closely reflects the reality. The exact place of birth (thus latitude) has not been recorded. However, the percentage of foreign-born students (that attended classes in Romanian) is likely to be (very) small. Thus it might be inferred that for most of them the latitude of their place of birth corresponds to that of Romania (i.e. roughly between 43 and 48°N).

Although we can speculate on potential RF that might explain these associations (see introduction) the present study was not designed to distinguish between several alternative hypotheses. However, as a whole our study suggests that urbanicity at birth increases the strength of the association and that climate might be important. Infectious hypotheses as for example those involving seasonal respiratory (e.g. influenza) or enteric viruses are compatible with these findings [12]. However, it seems too early to accept this hypothesis.

On the other hand, the present study suggests several methodological improvements that might advance our understanding and shed light on the RF associated with SoB. The use of homogeneous samples (age, urbanicity, latitude at birth) seems important. Also, future studies might benefit from the recent availability of data to include some of the hypothesized risk factors in the analyses (i.e. monthly records of temperature and days of sunshine, epidemiological data on infectious diseases like influenza etc.)

## Conclusion

The impact of SoB on schizotypy (and more generally on psychotic disorders) might be restricted to some specific conditions (latitude, climate). As a better understanding of the effective factors behind the association might advance our knowledge of risk factors for psychotic disorders the study of the association between SoB and schizotypy (and also of the factors that influence it) is worth pursuing.

## Supplementary Information

Below is the link to the electronic supplementary material.

Supplementary file1 (DOCX 42 KB)

## Data Availability

No datasets were generated or analysed during the current study.
