# Supplementary material for: Season of birth and schizotypy in a sample of undergraduate students
Source: Soc Psychiatry Psychiatr Epidemiol. 2024 Jul 9;60(2):319–28. doi: 10.1007/s00127-024-02719-w (PMC11839707; doi:10.1007/s00127-024-02719-w)
Supplement: Supplementary file 1 — Supplementary file1 (DOCX 42 KB) [file 127_2024_2719_MOESM1_ESM.docx]

# I. Social desirability items added to the SPQ-B questionnaire

(adapted from the Eysenck’s Lie scale)

When I take a decision or make a promise I always keep it, no matter how difficult it would be for me

I have never taken anything that was not mine, no matter how small that thing was

I have never blamed someone else when it was actually my fault

I’ve never said anything bad or unpleasant about someone else

I have never cheated when playing a game

#
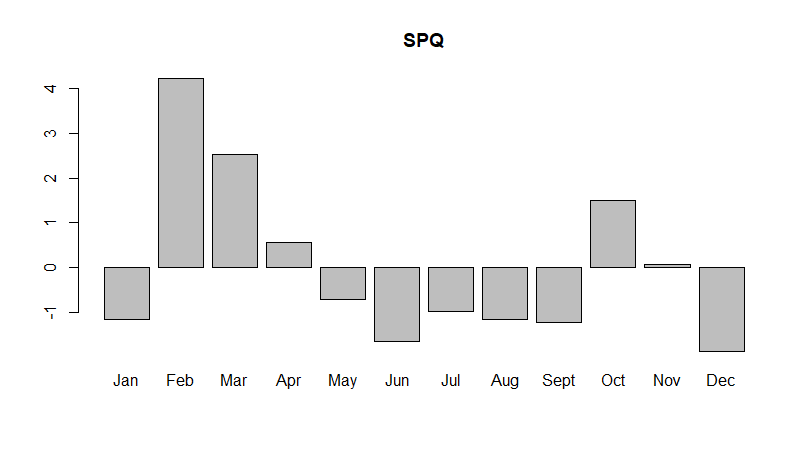
 II. Graphical representation of total SPQ scores

**Figure 1S.** Graphical representation of mean total SPQ score variation according to month of birth (represented as departure from the global mean)

# III. Supplementary analyses (using alternative definitions of season of birth)

## **1. Astronomic season** (winter from December 21st to March 20th, spring from March 21st to June 20th, summer from June 21st to September 20th and fall from September 21st to December 20th)

- 1. **Univariate**

|  |  | Winter | Spring | Summer | Fall |
| --- | --- | --- | --- | --- | --- |
|  | N | 130 | 124 | 122 | 108 |
| SPQ total | Mean | 56.41 | 54.48 | 53.84 | 54.55 |
|  | SD | 11.59 | 11.67 | 12.12 | 11.59 |
|  | t * | 1.74 | 0.43 | Ref | 0.46 |
|  | p * | 0.083 | 0.67 | Ref | 0.65 |
| Positive dim. | Mean | 25.91 | 25.44 | 24.76 | 25.41 |
|  | SD | 6.55 | 6.18 | 5.83 | 6.58 |
|  | t * | 1.45 | 0.84 | Ref | 0.78 |
|  | p * | 0.15 | 0.40 | Ref | 0.44 |
| Negative dim. | Mean | 16.35 | 15.50 | 15.89 | 15.70 |
|  | SD | 4.18 | 4.38 | 4.79 | 4.23 |
|  | t * | 0.83 | -0.70 | Ref | -0.33 |
|  | p * | 0.41 | 0.48 | Ref | 0.74 |
| Disorg. | Mean | 14.15 | 13.54 | 13.18 | 13.44 |
|  | SD | 4.18 | 3.92 | 4.11 | 4.22 |
|  | t * | 1.87 | 0.69 | Ref | 0.47 |
|  | p * | 0.063 | 0.49 | Ref | 0.64 |

## **Table 1S. Comparison of SPQ scores between astronomic seasons (summer = reference)**

| Category ^a^ | SPQ score (Mean) | t^b^ | p^c^ | Positive | t^b^ | p^c^ | Negative | t^b^ | p^c^ | Disorganization | t^b^ | p^c^ |
| --- | --- | --- | --- | --- | --- | --- | --- | --- | --- | --- | --- | --- |
| Winter | 56.41 | 1.77 | 0.077 | 25.91 | 1.11 | 0.27 | 16.35 | 1.46 | 0.15 | 14.15 | 1.81 | 0.071 |
| Other | 54.28 |  |  | 25.19 |  |  | 15.70 |  |  | 13.38 |  |  |

^a^ at risk = winter (21 December to 20 of March)

^b^ t statistics (Student’s t-test)

^c^ Probability of the null hypothesis (i.e. no difference between categories)

## **Table 2S. Univariate analyses astronomic winter vs the rest of the year**

- 1. **Multivariate**

|  |  | Tobacco  (ref No) | | Psychoactive subst. (ref No) | | Age | | Sex  (ref Male) | | At risk period ^a^ (ref No) | | Model | |
| --- | --- | --- | --- | --- | --- | --- | --- | --- | --- | --- | --- | --- | --- |
| SPQ dimension | Model^b^ | Reg. Coeff. | p-value | Reg. Coeff. | p-value | Reg. Coeff. | p-value | Reg. Coeff. | p-value | Reg. Coeff. | p-value | R² | p-value |
| SPQ total | 1 | *NA* | *NA* | *NA* | *NA* | -1.19 | 4.5*10^-5^ | -0.36 | 0.78 | 1.75 | 0.14 | 0.034 | 0.00018 |
|  | 2 | 1.27 | 0.26 | 1.37 | 0.0033 | -1.13 | 0.0033 | 0.42 | 0.74 | 1.46 | 0.22 | 0.052 | 1.2*10^-5^ |
| Positive dim. | 1 | *NA* | *NA* | *NA* | *NA* | -0.80 | 2.2*10^-7^ | 0.49 | 0.47 | 0.39 | 0.54 | 0.052 | 2.5*10^-6^ |
|  | 2 | 0.80 | 0.18 | 0.51 | 0.04 | -0.75 | 4.6*10^-6^ | 0.84 | 0.21 | 0.26 | 0.67 | 0.061 | 1.4*10^-6^ |
| Negative dim. | 1 | *NA* | *NA* | *NA* | *NA* | -0.08 | 0.46 | 0.24 | 0.62 | 0.61 | 0.18 | -0.00017 | 0.41 |
|  | 2 | -0.33 | 0.45 | 0.46 | 0.010 | -0.13 | 0.26 | 0.34 | 0.49 | 0.55 | 0.22 | 0.010 | 0.080 |
| Disorg. | 1 | *NA* | *NA* | *NA* | *NA* | -0.30 | 0.0028 | -1.08 | 0.01 | 0.75 | 0.07 | 0.031 | 0.00046 |
|  | 2 | 0.80 | 0.044 | 0.41 | 0.013 | -0.25 | 0.020 | -0.77 | 0.087 | 0.64 | 0.12 | 0.049 | 2.2*10^-5^ |

^a^ at risk = winter (astronomic 21 December to 20 of March)

^b^ Model 1 includes only demographic variables (Age and Sex) and Season of Birth

Model 2 includes demographic variables (Age and Sex), tobacco, psychoactive substances and Season of Birth

## **Table 3S. Multivariate analyses with winter (astronomic) as the risk period**

## **2. Meteorologic season** (winter from December 1st to end of February, spring from March 1st to May 31st, summer from Jun 1st to August 31st and fall from September 1st to December 31st)

**2.1. Univariate**

|  |  | Winter | Spring | Summer | Fall |
| --- | --- | --- | --- | --- | --- |
|  | N | 130 | 124 | 122 | 108 |
| SPQ total | Mean | 55.18 | 55.54 | 53.58 | 55.07 |
|  | SD | 11.49 | 11.47 | 12.47 | 11.59 |
|  | t * | 1.06 | 1.34 | Ref | 0.97 |
|  | p * | 0.29 | 0.18 | Ref | 0.34 |
| Positive dim. | Mean | 25.91 | 25.44 | 24.76 | 25.41 |
|  | SD | 6.55 | 6.18 | 5.83 | 6.58 |
|  | t * | 1.45 | 0.84 | Ref | 0.78 |
|  | p * | 0.15 | 0.40 | Ref | 0.44 |
| Negative dim. | Mean | 16.35 | 15.50 | 15.89 | 15.70 |
|  | SD | 4.18 | 4.38 | 4.79 | 4.23 |
|  | t * | 0.83 | -0.70 | Ref | -0.33 |
|  | p * | 0.41 | 0.48 | Ref | 0.74 |
| Disorg. | Mean | 14.15 | 13.54 | 13.18 | 13.44 |
|  | SD | 4.18 | 3.92 | 4.11 | 4.22 |
|  | t * | 1.87 | 0.69 | Ref | 0.47 |
|  | p * | 0.063 | 0.49 | Ref | 0.64 |

## **Table 4S. Comparison of SPQ scores between meteorologic seasons (summer - reference)**

| Category ^a^ | SPQ score (Mean) | t^b^ | p^c^ | Positive | t^b^ | p^c^ | Negative | t^b^ | p^c^ | Disorganization | t^b^ | p^c^ |
| --- | --- | --- | --- | --- | --- | --- | --- | --- | --- | --- | --- | --- |
| Winter | 55.18 | 0.35 |  | 25.37 | -0.033 |  | 15.98 | 0.31 |  | 13.82 | 0.72 |  |
| Other | 54.74 |  | 0.73 | 25.39 |  | 0.97 | 15.84 |  | 0.76 | 13.51 |  | 0.47 |

^a^ at risk = winter (astronomic: 21 December to 20 of March; meteorologic: December to February)

^b^ t statistics (Student’s t-test)

^c^ Probability of the null hypothesis (i.e. no difference between categories)

## **Table 5S. Univariate analyses meteorologic winter vs the rest of the year**

- 1. **Multivariate**

|  |  | Tobacco  (ref No) | | Psychoactive subst. (ref No) | | Age | | Sex  (ref Male) | | At risk period ^a^ (ref No) | | Model | |
| --- | --- | --- | --- | --- | --- | --- | --- | --- | --- | --- | --- | --- | --- |
| SPQ dimension | Model^b^ | Reg. Coeff. | p-value | Reg. Coeff. | p-value | Reg. Coeff. | p-value | Reg. Coeff. | p-value | Reg. Coeff. | p-value | R² | p-value |
|  | 1 | *NA* | *NA* | *NA* | *NA* | -1.22 | 2.8*10^-5^ | -0.19 | 0.88 | 0.24 | 0.84 | 0.030 | 0.00050 |
|  | 2 | 1.34 | 0.24 | 1.40 | 0.00 | -1.15 | 0.00 | 0.59 | 0.64 | 0.02 | 0.99 | 0.049 | 2.4*10^-5^ |
|  | 1 | *NA* | *NA* | *NA* | *NA* | -0.81 | 1.5*10^-7^ | 0.53 | 0.42 | -0.17 | 0.79 | 0.052 | 3.0*10^-6^ |
|  | 2 | 0.81 | 0.18 | 0.52 | 0.037 | -0.76 | 3.7*10^-6^ | 0.89 | 0.19 | -0.26 | 0.69 | 0.061 | 1.4*10^-6^ |
|  | 1 | *NA* | *NA* | *NA* | *NA* | -0.092 | 0.40 | 0.29 | 0.54 | 0.12 | 0.80 | -0.0037 | 0.75 |
|  | 2 | -0.30 | 0.49 | 0.47 | 0.0085 | -0.14 | 0.23 | 0.40 | 0.41 | 0.047 | 0.92 | 0.0070 | 0.14 |
|  | 1 | *NA* | *NA* | *NA* | *NA* | -0.32 | 0.0019 | -1.02 | 0.021 | 0.30 | 0.49 | 0.025 | 0.0017 |
|  | 2 | 0.83 | 0.037 | 0.41 | 0.012 | -0.26 | 0.017 | -0.70 | 0.12 | 0.23 | 0.60 | 0.045 | 5.6*10^-5^ |

^a^ at risk = winter (meteorologic December to February)

^b^ Model 1 includes only demographic variables (Age and Sex) and Season of Birth

Model 2 includes demographic variables (Age and Sex), tobacco, psychoactive substances and Season of Birth

## **Table 6S. Multivariate analyses with winter (meteorologic) as risk period**
